# Body Composition Changes over Multiple Academic Years in Female Collegiate Soccer Players

**DOI:** 10.3390/jfmk5040072

**Published:** 2020-09-28

**Authors:** Austin Katona, Caroline Riewe, Angela Bruzina, Nicholas J Ollberding, Mary Ankrom, Jon Divine, Robert Mangine, Abigail Peairs

**Affiliations:** 1 Department of Rehabilitation, Exercise, and Nutrition Sciences, University of Cincinnati, Cincinnati, OH 45267, USA; katonaaj@mail.uc.edu (A.K.); riewece@mail.uc.edu (C.R.); bruzinas@ucmail.uc.edu (A.B.); 2 Division of Biostatistics and Epidemiology, Cincinnati Children’s Hospital Medical Center, Cincinnati, OH 45229, USA; nicholas.ollberding@cchmc.org; 3 Department of Pediatrics, College of Medicine, University of Cincinnati, Cincinnati, OH 45267, USA; 4 Department of Athletics, University of Cincinnati, Cincinnati, OH 45267, USA; ankromme@ucmail.uc.edu (M.A.); divinej@ucmail.uc.edu (J.D.); manginre@ucmail.uc.edu (R.M.); 5 NovaCare Rehabilitation, Mechanicsburg, PA 17055, USA

**Keywords:** body composition, female athlete, soccer, sports nutrition, athlete assessment, longitudinal assessment

## Abstract

Body composition plays a key role in overall health and sports performance and its assessment is an important part of many athletic programs. The purpose of this study was to describe longitudinal changes in body composition for collegiate female soccer players in order to provide data to inform future training and nutrition interventions for this population. A linear mixed-model (LMM) approach was used to analyze four years of pre- and post-season body composition data, including total mass, fat-free mass (FFM), fat mass, and body fat percentage (%BF) for 49 athletes. Athletes gained an average of 0.5 kg FFM during the season (*p* < 0.05) and increased total mass, FFM, fat mass, and %BF (2.5 kg, 1.1 kg, 1.7 kg, and 1.7%, respectively; *p* < 0.05) over four years. Freshmen experienced a 1.5 kg gain in total mass pre- to post-season (*p* < 0.05), while no changes in total mass or body composition were seen in other grade levels. Gains in %BF during the off season between Freshman and Sophomore years represented negative changes in body composition that should be addressed further. These results can help interdisciplinary athlete care teams optimize training programs in this population by understanding what changes are expected over multiple years. Normalizing these changes may also help the promotion of realistic body composition goals and the development of positive training and dietary habits.

## 1. Introduction

Body composition is one of many factors that influences athletes’ sports performance and overall health and is commonly assessed as part of an athlete’s standard of care while competing at the collegiate level [1,2,3,4,5]. Body composition is related to many different health outcomes, including altered bone remodeling and endocrine functioning at low levels of body fat, and increased risk of metabolic comorbidities, such as dyslipidemia and insulin resistance with excessive body fat [1,2,6]. Although these outcomes are important to consider with athletes, as they could affect overall health and athletic performance, body composition assessment in sports medicine is often focused on much smaller variations in body composition, which are thought to affect athletic performance and injury risk [1,2,3,6]. In sports science research, it is commonly accepted that having higher fat-free mass (FFM) and a lower body fat percentage (%BF) helps to optimize an athlete’s potential for speed and power [1,2,3,7,8]. Therefore, assessing body composition in athletes can give insight into both overall health and sports performance.

Collegiate athletes are exposed to rigorous training schedules in order to maximize their growth in strength, speed, and skill. However, athletes are often reported to consume inadequate energy and female athletes, in particular, have increased potential for developing disordered eating habits [9,10,11]. These challenges may make changes in body composition especially hard to predict for female athletes. Examining changes in body composition over a season is useful because it reflects both the effects of training, as well as the nutritional status of the athlete [1,2,3,4,5,12]. Additionally, the assessment of body composition across several years provides both athletes and interdisciplinary care teams (i.e., athletic trainers, strength and conditioning coaches, sports dietitians) a sense of expected body composition changes over an athlete’s collegiate career and provides training personnel pertinent feedback regarding strength and conditioning programs, as well as athlete’s dietary behaviors [2,3,5,13,14]. This may also help increase the acceptance of nutrition protocols designed to support growth and development of the athletes by enhancing athletes’ understanding of body composition changes associated with those behaviors. It is important to note that body composition differs by sport and sex, so establishing normative data specific to sport and sex is vital for athlete care teams to monitor athlete health and effectiveness of training programs [3,5].

Few studies have reported longitudinal changes in body composition in female athletes, especially collegiate soccer players [1,2,3,15]. In those studies that have assessed body composition changes in female soccer players, there are mixed data regarding changes pre- to post-season, where some studies reported no changes [1,16], while another showed decreases in FFM [17]. There is even less information across multiple seasons with only one study assessing body composition across three seasons [1], with no significant changes in total mass, %BF, or FFM noted over the time period studied.

The objective of this study was to describe body composition changes for female collegiate National Collegiate Athletic Association (NCAA) Division 1 soccer players both pre- to post-season and between academic grade levels, over the course of multiple competitive seasons. Entry into college athletics exposes the athlete to new training stimuli with higher physiological demands, so we predict the largest changes in body composition to occur over the freshman year, in comparison to later years. It is also hypothesized that the athletes would continue to grow over the years and experience increases in body mass, including both FFM and %BF.

## 2. Materials and Methods

### 2.1. Protocol

This study consisted of a secondary data analysis of body composition measurements of female NCAA Division 1 soccer athletes from a single institution, taken as part of standard of care from 2016 to 2019. The dataset included pre- and post-season total mass, %BF, and FFM measures for 49 female collegiate athletes over the course of four academic years. Since the athletes participated in a Fall sport, pre-season assessments were typically completed in July or August each year, before the competitive soccer seasons began, and post-season assessments were completed in November after the conclusion of the competitive soccer season. Due to utilization of previously collected de-identified data, this study was determined to be not human-subjects research by the Institutional Review Board at the University of Cincinnati.

### 2.2. Participants

There was a total of 34 freshmen (FR), 26 sophomore (SO), 22 junior (JR), and 14 senior (SR) individual data points, with 20 participants contributing one year of data, 15 participants with 2 years, 10 participants with 3 years, and 4 participants with 4 years of body composition data. Each individual contributed both pre- and post-season measurements for each participating year. Training for these athletes typically started with 2 weeks of pre-season strength and conditioning from the end of July to the middle of August and usually included 2 training sessions per day. This was followed by the 4 to 5-week non-conference season with about 2 games per week and the conference season, which was also 4 to 5 weeks, including about 10 games. Energy expenditure in this phase of the season was more variable for individual athletes based on the amount of time played during games. In the winter/spring post-season, the athletes trained up to 20 h a week until the end of April, then were given optional, self-initiated work outs in the summer off season that lasted from the end of April to the end of July.

### 2.3. Body Composition Assessment

Total mass was measured using a standard scale (COSMED, Concord, CA, USA), height was measured using a wall mounted stadiometer (Height-Rite, model 225, SECA, Chino, CA, USA) and %BF and FFM were assessed by air-displacement plethysmography (ADP) using the BodPod (COSMED, Concord, CA, USA; software version 2.30). Validity tests show that ADP may overestimate %BF in female athletes when compared to Dual-energy X-ray absorptiometry (DXA) [18], and this should be taken into consideration for future comparisons.

### 2.4. Statistical Analysis

A restricted maximum-likelihood linear mixed model (LMM) was used to assess differences in body composition over the course of a competitive soccer season and between academic grade levels [19]. A mixed model approach was used to account for the repeated measure nature of the data and varying number of data points for each participant. Outcomes assessed were total mass, %BF, fat mass, and FFM. Time point (pre-season or post-season) and academic grade level were included as fixed-effect terms. A random subject-specific intercept was included to account for repeated measures within athletes. Examination of Q–Q and residual plots indicated that assumptions for the LMMs were met. LMMs for each outcome included grade level and time point as factor variables and an interaction term between grade level and time point. The least-squares means test with Tukey adjustment provided pairwise comparisons between grade levels, time points, and time points for each grade level [20]. The Kenward–Roger approximation to model degrees of freedom was used to obtain *p*-values. A *p*-value of *p* ≤ 0.05 was considered statistically significant in all analyses, which were conducted using R v3.6.3 [21], including the tidyverse v.1.3.1 [22], readxl v.1.3.1 [23], lme4 v1.1.21 [19], lmerTest v3.1.0 [24], emmeans v1.4.1 [20], and broom v0.5.2 packages [25].

## 3. Results

Likelihood ratio tests revealed that the grade level factor was significant for all outcomes examined (*p* < 0.05), while the time point factor was significant for FFM (df = 1, Χ^2^ = 6.39, *p* = 0.011) and the interaction term between grade level and time point was significant for total mass (df = 3, Χ^2^ = 11.09, *p* = 0.011). Since the data were analyzed using LMMs, statistically significant differences were determined using contrasts of the adjusted means.

### 3.1. Between Grade Levels

**Figure 1:**
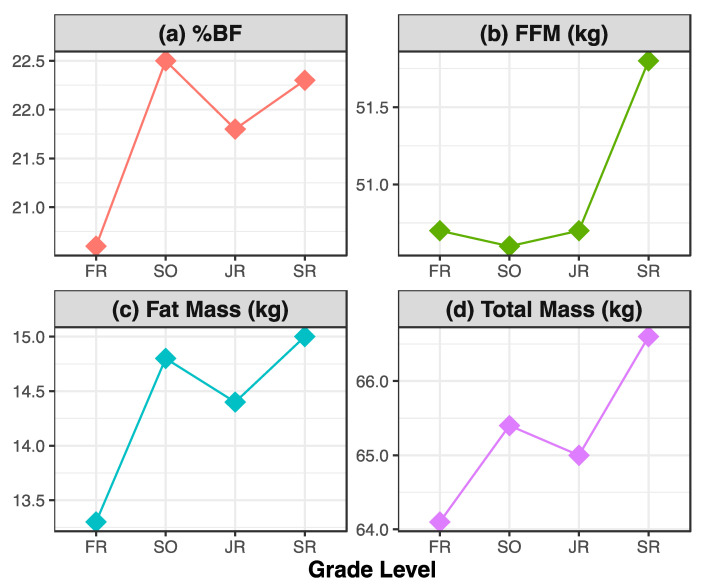
Average adjusted body composition measures by grade level, averaged over time point (pre- and post-season). (**a**) average percent body fat by grade level; (**b**) average fat-free mass by grade level, in kg; (**c**) average fat mass by grade level, in kg; (**d**) average total mass by grade level, in kg.

Observed and adjusted means for total mass, %BF, fat mass, and FFM overall and across grade levels are presented in Table 1. SO and SR had higher total mass than FR, when averaged over the competitive soccer season (Mean Difference (MD) = 1.25 kg, *p* = 0.021 and 2.52 kg, *p* < 0.001, respectively). Additionally, SR had higher total mass than JR (MD = 1.62 kg, *p* = 0.027). SO, JR, and SR had higher %BF (MD = 1.89%, *p* = 0.001, 1.26%, *p* = 0.037, and 1.75%, *p* = 0.022, respectively) and fat mass (MD = 1.49 kg, *p* = 0.0001, 1.07 kg, *p* = 0.023, and 1.75 kg, *p* = 0.002, respectively) than FR. SR FFM measurements were higher than both SO and JR values, by 1.15 kg (*p* = 0.034) and 1.06 kg (*p* = 0.039), respectively. Each body composition measure tended to increase over the course of a four-year collegiate soccer career for these female athletes, with statistically significant increases in total mass, %BF, fat mass, and FFM during that time period. Figure 1 depicts the trajectory of body composition changes over a four-year period, averaged between adjusted pre- and post-season measurements.

### 3.2. Pre- to Post-Season

Observed and adjusted means from the LMMs for body composition changes from pre- to post-season are presented in Table 2. Overall, there was a statistically significant increase in FFM from pre- to post-season (MD = 0.47 kg, *p* = 0.024), but no changes in other body composition measures. FR experienced an average gain of 1.47 kg in total mass (*p* = 0.048), but no statistically significant changes in other measures. Of note, however, FR was the only grade level to exhibit an average increase in %BF and fat mass from pre- to post-season, although it was not significant in the LMM. There were no changes in total mass, %BF, fat mass, or FFM from pre- to post-season for SO, JR, or SR.

Pre-season SO and SR had higher total mass, fat mass, and %BF that pre-season FR (*p* < 0.05), meaning that SO and SR started their competitive soccer seasons at higher total mass, fat mass, and %BF than FR, on average. Post-season SR had higher total mass and FFM than pre-season FR (*p* < 0.05), meaning that, on average, athletes gained a statistically significant amount of total mass and FFM between the start of their first competitive collegiate soccer season and the end of their fourth competitive season. Pre-season SO had higher %BF and fat mass than post-season FR (*p* < 0.05), indicating a statistically significant average increase in %BF and fat mass during the off season between FR and SO years. There were no changes in %BF or fat mass between SO and JR years or between JR and SR years. Additionally, there were no changes in FFM between consecutive competitive seasons, although non-statistically significant decreases in FFM were observed between the FR and SO years and the SO and JR years.

## 4. Discussion

The current study provided body composition data for female NCAA Division 1 collegiate soccer players pre- and post-season over multiple years. The athletes in this study had higher reported total mass (65.4 vs. 61.8–65 kg) and FFM (51.2 vs. 44.4–48 kg) compared to other studies describing female collegiate soccer players [1,4,5,16,17]. %BF measurements for athletes in this study were lower than in four other studies (21.7% vs. 24.1–27.7%) [1,4,5,17], but higher than measurements in one study (21.7% vs. 16.3%) [16]. It is important to note that these studies used different body composition assessment methods (i.e., DXA or hydrostatic weighing), and therefore caution must be taken when comparing results.

Overall, the only change observed in body composition between pre- and post-season was a 0.5 kg increase in FFM (*p* < 0.05). Similar increases in FFM have been observed in adolescent female soccer players, although these gains were accompanied by decreases in %BF [14]. The results of the current study contradict other previous research, with two studies reporting no body composition changes [1,16], and another reporting decreases in %BF and fat mass but the maintenance of FFM, all in female collegiate soccer players [12]. Another study that analyzed athletes based on play time actually reported a decrease in FFM in female collegiate soccer players, although this decrease was only significant in non-starters [17]. Differences could again be attributed to training program specifics, as well as populations studied. The increase in FFM over the competitive season seen in the current study, especially in FR, would be expected due to the high level of training during the season. However, the use of air-displacement plethysmography, a method that uses a two-compartment body composition model, limits any conclusions made about skeletal muscle changes because it treats muscle as FFM in combination with bone and body water [2].

It is also important to compare individual grade levels, as previous research has shown that within-person variation could be attributed to grade level differences in play time and physiological adaptations [5,26]. When considering specific time periods, FR experienced an increase in total mass between pre- and post-season measurements but did not experience any notable changes in body composition. The hypothesis that FR would experience the largest changes in body composition cannot be supported by the results of the current analysis, although it can be concluded that FR gained the most total mass pre- to post-season. No other studies on collegiate female soccer players included pre- to post-season body composition data specific to academic grade level, which further highlights the need for more quality longitudinal research in this population.

When comparing results of the current study to those obtained from examination of female athletes in other sports, one study reported significant pre- to post-season increases in FFM for swimming, track, and volleyball, and significant decreases in %BF in swimming, track, and basketball [1]. Another study reported no changes in body composition for female basketball players over the course of the season [2], with yet another study reporting an increase in %BF and no change in FFM for female ice hockey players [27]. Decreases in FFM were seen in female collegiate softball players in a study by Peart, et al. [3], although softball is arguably a much different sport than soccer in terms of training and energy requirements. The variability in body composition changes among collegiate sports highlights the importance of establishing normative data for each specific sport in order to optimize training outcomes.

Due to normal growth and development, as well as the effects of multiple years of athletic training, it was hypothesized that female athletes would gain total mass, fat mass, and FFM during their collegiate soccer careers. By their SR year, athletes had gained total mass, which included increases in fat mass, %BF, and FFM. When looking at values averaged between pre- and post-season measurements, fat mass and %BF increased between FR and SO year (*p* < 0.05), and then remained steady through the SR year. Assessing pre- and post-season measurements separately shows that SO and SR pre-season %BF and fat mass measurements were higher than FR pre-season measurements (*p* < 0.05). This means that SO and SR generally started their competitive soccer seasons with more fat mass than FR. FFM did not change between FR and JR years but increased between JR and SR years. SR post-season FFM was higher than FR pre-season FFM (*p* < 0.05), which supports the conclusion that these athletes gained FFM over their collegiate soccer careers. The results of the current study are different than the only other study on longitudinal body composition changes in female soccer athletes, in which participants had no change in total mass, fat mass, %BF, or FFM over 3 years [1]. This same previous study, which included longitudinal data for multiple sports, concluded that female swimmers and volleyball players showed longitudinal increases in FFM, while basketball players had increases in total mass, fat mass, and %BF over 3 years [1]. Another study of female collegiate basketball players reported no changes in total mass, fat mass, or %BF over 3 years, but statistically significant increases in FFM between SO and JR year [2]. This is similar to the current study, in which there were no changes in pre- to post-season averaged FFM until later in the athlete’s collegiate career, with increases between JR and SR years. While total mass, %BF, and fat mass all increased between FR and SO years, FFM remained the same. This incited further investigation into why this trend was occurring.

A key to understanding longitudinal changes in body composition for collegiate athletes is to consider both on- and off-season changes [12]. Off-season changes in the current study occurred between the end of one competitive season (November) and the start of the next pre-season (July/August), including both spring training and summer off-season periods. When taking a closer look at changes during this time, raw data showed a non-significant decrease in FFM in the off season after FR year (0.5 kg observed, 0.7 kg adjusted). Although it cannot be concluded that FFM decreased during this time, this explains why there is not an increase in time-point averaged FFM between FR and SO years. This is also surprising because the off-season changes included a period of increased strength and conditioning during the spring, where increases in FFM would be expected. A 0.81 kg off-season loss of FFM (*p* < 0.05) was also observed in a study on female collegiate basketball players [2]. Although not present in the current study, any reductions in FFM should be of concern to the athlete care team because they may be associated with changes in athletic performance, nutrition habits, and bone mineral density, as well as increased injury risk upon return to play [2,3,28,29].

Upon further examination of the off-season changes, the LMM also revealed that there was an increase in fat mass and %BF between FR and SO years (*p* < 0.05). This off-season gain in fat mass has been reported by another group studying changes in body composition and bone density in female collegiate soccer players [17]. Additionally, a recent review of off-season changes in soccer players found small increases in %BF and large decreases in FFM in studies of professional soccer players [28]. Contradictory to these results, a decrease in %BF was observed in a study on female collegiate NCAA Division II athletes [12], although off-season training differences could affect comparisons. Off-season gain in %BF seen in the current study constitutes a negative change in body composition that could be detrimental to an athlete on multiple levels, and therefore must be addressed [1]. However, without dietary intake data or detailed off-season training information, it is difficult to determine why these negative body composition changes are occurring. Future studies in this area should collect this information to determine the etiology of the changes so that appropriate interventions can be developed to target and prevent those behaviors. Regardless, highlighting regressions in body composition may give vital feedback to coaching and sports nutrition staff in order to help athletes make beneficial changes to off-season practices.

Longitudinal body composition assessment is a beneficial aspect of collegiate athletic programs because it provides feedback on the strength and conditioning programs and nutritional statuses of athletes [1,2,3,4,5,12]. Previous studies argue that comparing body composition changes between males and females and even athletes and non-athletes is difficult and may skew conclusions [5,14]. Data specific to sport and sex is important to collect for comparison of body composition changes because of the high variability in %BF and FFM distribution between males and females, and between sports due to sport-specific adaptations [2,3,5,15,26,27,30]. Therefore, the current study gives an initial look into how body composition may change in female collegiate soccer players at this institution, over multiple years. This could provide a comparison for athletic trainers and strength and conditioning coaches to track the development of future athletes in order to optimize training programs and initiate interventions when athletes are experiencing negative changes. However, athletes should also be compared to their own baseline training and body composition data due to inevitable within-person variation [5].

Established data for longitudinal body composition changes would likely be helpful for an athlete’s interdisciplinary care team but may also be beneficial for the athlete themselves. Participation in collegiate athletics is accompanied by changes in body composition and energy demands that continually fluctuate [3]. On top of social, behavioral, and dietary changes that occur when entering a college environment, collegiate athletes experience greater physical changes from heightened energy demands and intense training schedules [10,31,32]. Pressure from coaches to attain a certain body weight and composition, as well as the desire to achieve athletic performance goals could lead to unhealthy dietary habits and possibly the development of disordered eating habits, which is known to be common in female athletes [9,10,11]. Having sport- and sex-specific body composition data to reference could help normalize changes seen in female athletes, especially increases in total mass and %BF over time, which could reduce desire to strive for an unrealistic physique. Understanding this as a normal pattern of growth and development could also help coaches and trainers effectively guide athletes toward more realistic body composition goals, helping to prevent the development of unhealthy dietary habits and body dysmorphia. Coaches and trainers play a vital role in shaping athlete’s views on body composition changes and overall body acceptance and should therefore prevent practices that may lead to disordered eating [33]. This also highlights the importance of having an interdisciplinary team to help care for and train the athlete. The results of the current study describe longitudinal body composition changes in female collegiate soccer players at a single institution, and this data may be beneficial to both athletes and their care team in an aim to optimize health and performance outcomes for this population.

This study adds to the limited literature on body composition changes in female collegiate soccer players, but it is not without limitations. Age and race were not included in the dataset, and both could confound body composition comparisons person to person because of variability in body composition across ages and races. Additionally, the sample size for this dataset was small, especially for upperclassmen (JR and SR), which could have affected the results of the LMM and therefore the trends that were observed. The absence of play time and training data could have potentially mitigated differences in body composition changes between athletes based on how much they played. The use of athletes from a single institution decreases the generalizability of the results. The use of ADP rather than DXA for body composition assessment restricts the generalizability of the results and limits the possible comparisons to studies of similar populations. With these limitations, there are also strengths to the current study. The dataset included multiple cohorts of athletes, so differences from year to year were able to be assessed in multiple groups rather than a single group over time. This prevented the data from being skewed by the unique characteristics of a single team or recruitment cohort. Another strength to the current study is the linear mixed model approach, which accounted for variation and the repeated-measure nature of the data. Future research should include a larger sample size of athletes and compare body composition changes to nutritional intake and performance outcomes. More frequent measurements should also be taken to account for fluctuations in body composition and energy balance in different phases of a competitive soccer season.

## 5. Conclusions

This study reported statistically significant increases in FFM between pre- and post-season and longitudinal increases in all body composition measures for female collegiate soccer players. Analysis of off-season changes revealed increases in %BF between FR and SO year, which may indicate the need for development of more focused off-season training and nutrition practices. Longitudinal body composition data can be used to give the interdisciplinary athlete care team vital feedback on training and nutrition practices, but also help mitigate detraining during the off season by informing new approaches to that often-neglected time of the year. Using established body composition data that is specific to sport and sex in tandem with comparing athletes against their own baseline data could prove helpful for tracking the growth of athletes over an extended period of time and help improve understanding of expected changes, optimizing the physical and mental health of these athletes.

## Figures and Tables

**Table 1:** Observed and adjusted means for body composition measures overall and across grade levels, averaged over a competitive soccer season *.

	Total Mass (kg)	%BF	Fat Mass (kg)	FFM (kg)
Overall	65.4 ± 1.0	21.7 ± 0.6	14.3 ± 0.5	51.2 ± 0.8
	(65.3 ± 1.0)	(21.8 ± 0.5)	(14.3 ± 0.5)	(51.0 ± 0.8)
Freshman	63.7 ± 0.9	20.4 ± 0.5	13.0 ± 0.4	50.7 ± 0.8
	(64.1 ± 1.0)	(20.6 ± 0.6)	(13.3 ± 0.5)	(50.7 ± 0.8)
Sophomore	65.1 ± 1.0	22.1 ± 0.6	14.4 ± 0.5	50.7 ± 0.7
	(65.4 ± 1.1) ^	(22.5 ± 0.6) ^	(14.8 ± 0.5) ^	(50.6 ± 0.8)
Junior	66.5 ± 1.2	21.9 ± 0.6	14.7 ± 0.5	51.8 ± 0.8
	(65.0 ± 1.1)	(21.8 ± 0.6)^	(14.4 ± 0.5) ^	(50.7 ± 0.8)
Senior	68.5 ± 1.1	23.5 ± 0.5	16.2 ± 0.5	52.4 ± 0.8
	(66.6 ± 1.1) ^†	(22.3 ± 0.7) ^	(15.0 ± 0.6) ^	(51.8 ± 0.8) †§

* Observed mean ± SE (Adjusted mean ± SE). ^ Significantly different than Freshman, *p* < 0.05. § Significantly different than Sophomore, *p* < 0.05. † Significantly different than Junior, *p* < 0.05. %BF = percent body fat; FFM = fat-free mass.

**Table 2:** Observed and adjusted means for pre- and post-season body composition measures.*

	Total Mass (kg)	%BF	Fat Mass (kg)	FFM (kg)
Overall (Pre)	65.3 ± 1.0	21.8 ± 0.6	14.4 ± 0.5	50.9 ± 0.8
	(65.2 ± 1.0)	(22.0 ± 0.6)	(14.5 ± 0.5)	(50.7 ± 0.8)
Overall (Post)	65.6 ± 1.0	21.5 ± 0.5	14.2 ± 0.5	51.4 ± 0.8
	(65.4 ± 1.0)	(21.6 ± 0.6)	(14.2 ± 0.5)	(51.2 ± 0.8) ^
Freshman (Pre)	63.0 ± 0.9	20.2 ± 0.6	12.8 ± 0.4	50.2 ± 0.8
	(63.4 ± 1.1)	(20.4 ± 0.6)	(13.0 ± 0.5)	(50.3 ± 0.8)
Freshman (Post)	64.5 ± 0.9	20.6 ± 0.5	13.3 ± 0.4	51.1 ± 0.8
	(64.9 ± 1.1) ^	(20.8 ± 0.6)	(13.6 ± 0.5)	(51.2 ± 0.8)
Sophomore (Pre)	65.5 ± 1.0	22.6 ± 0.6	14.9 ± 0.5	50.6 ± 0.8
	(65.8 ± 1.1) †	(23.0 ± 0.6) †§	(15.2 ± 0.6) †§	(50.5 ± 0.8)
Sophomore (Post)	64.6 ± 1.0	21.6 ± 0.5	14.0 ± 0.4	50.8 ± 0.7
	(64.9 ± 1.1)	(22.0 ± 0.7)	(14.4 ± 0.6)	(50.7 ± 0.8)
Junior (Pre)	66.5 ± 1.2	22.1 ± 0.6	14.9 ± 0.6	51.6 ± 0.8
	(64.9 ± 1.1)	(22.0 ± 0.7)	(14.5 ± 0.6)	(50.5 ± 0.8)
Junior (Post)	66.6 ± 1.3	21.8 ± 0.5	14.6 ± 0.5	52.0 ± 0.9
	(65.1 ± 1.1)	(21.7 ± 0.7)	(14.2 ± 0.6)	(50.9 ± 0.8)
Senior (Pre)	68.6 ± 1.1	24.0 ± 0.6	16.5 ± 0.5	52.1 ± 0.8
	(66.7 ± 1.2) †	(22.8 ± 0.8) †	(15.4 ± 0.7) †	(51.5 ± 0.9)
Senior (Post)	68.5 ± 1.1	23.0 ± 0.5	15.8 ± 0.5	52.6 ± 0.8
	(66.6 ± 1.2) †	(21.9 ± 0.8)	(14.7 ± 0.7)	(52.0 ± 0.9) †

* Observed mean ± SE (Adjusted mean ± SE). ^ Significantly different than Pre-season, *p* < 0.05. † Significantly different than Freshman (Pre), *p* < 0.05. § Significantly different than Freshman (Post), *p* < 0.05. %BF = percent body fat; FFM = fat-free mass; Pre = pre-season; Post = post-season.

## References

[1] Stanforth P.R., Crim B.N., Stanforth D., Stults-Kolehmainen M.A. (2014). Body composition changes among female NCAA division 1 athletes across the competitive season and over a multiyear time frame. J. Strength Cond. Res..

[2] Fields J.B., Merrigan J.J., White J.B., Jones M.T. (2018). Seasonal and Longitudinal Changes in Body Composition by Sport-Position in NCAA Division I Basketball Athletes. Sports.

[3] Peart A., Wadsworth D., Washington J., Oliver G. (2019). Body Composition Assessment in Female National Collegiate Athletic Association Division I Softball Athletes as a Function of Playing Position Across a Multiyear Time Frame. J. Strength Cond. Res..

[4] Dobrosielski D.A., Leppert K.M., Knuth N.D., Wilder J.N., Kovacs L., Lisman P.J. (2019). Body Composition Values of Division 1 Female Athletes Derived from Dual-Energy X-Ray Absorptiometry. J. Strength Cond. Res..

[5] Sanfilippo J., Krueger D., Heiderscheit B., Binkley N. (2019). Dual-Energy X-Ray Absorptiometry Body Composition in NCAA Division I Athletes: Exploration of Mass Distribution. Sports Health.

[6] Thomas D.T., Erdman K.A., Burke L.M. (2016). American College of Sports Medicine Joint Position Statement. Nutrition and Athletic Performance. Med. Sci. Sports Exerc..

[7] Dengel D.R., Keller K.A., Stanforth P.R., Oliver J.M., Carbuhn A., Bosch T.A. (2020). Body Composition and Bone Mineral Density of Division 1 Collegiate Track and Field Athletes, a Consortium of College Athlete Research (C-CAR) Study. J. Clin. Densitom..

[8] Abe T., Kawamoto K., Dankel S.J., Bell Z.W., Spitz R.W., Wong V., Loenneke J.P. (2020). Longitudinal associations between changes in body composition and changes in sprint performance in elite female sprinters. Eur. J. Sport Sci..

[9] Shriver L.H., Betts N.M., Wollenberg G. (2013). Dietary intakes and eating habits of college athletes: Are female college athletes following the current sports nutrition standards?. J. Am. Coll. Health.

[10] Coelho G.M., Gomes A.I., Ribeiro B.G., Soares Ede A. (2014). Prevention of eating disorders in female athletes. Open Access J. Sports Med..

[11] Cosh S., Crabb S., LeCouteur A., Kettler L. (2012). Accountability, monitoring and surveillance: Body regulation in elite sport. J. Health Psychol..

[12] Peart A.N., Nicks C.R., Mangum M., Tyo B.M. (2018). Evaluation of Seasonal Changes in Fitness, Anthropometrics, and Body Composition in Collegiate Division II Female Soccer Players. J. Strength Cond. Res..

[13] Walker E.J., Aughey R.J., McLaughlin P., McAinch A.J. (2020). Seasonal Change in Body Composition and Physique of Team Sport Athletes. J. Strength Cond. Res..

[14] Lesinski M., Prieske O., Helm N., Granacher U. (2017). Effects of Soccer Training on Anthropometry, Body Composition, and Physical Fitness during a Soccer Season in Female Elite Young Athletes: A Prospective Cohort Study. Front. Physiol..

[15] Milanović Z., Sporiš G., James N., Trajković N., Ignjatović A., Sarmento H., Trecroci A., Mendes B.M.B. (2017). Physiological Demands, Morphological Characteristics, Physical Abilities and Injuries of Female Soccer Players. J. Hum. Kinet..

[16] Clark M., Reed D.B., Crouse S.F., Armstrong R.B. (2003). Pre- and post-season dietary intake, body composition, and performance indices of NCAA division I female soccer players. Int. J. Sport Nutr. Exerc. Metab..

[17] Minett M.M., Binkley T.B., Weidauer L.A., Specker B.L. (2017). Changes in body composition and bone of female collegiate soccer players through the competitive season and off-season. J. Musculoskelet Neuronal Interact..

[18] Vescovi J.D., Hildebrandt L., Miller W., Hammer R., Spiller A. (2002). Evaluation of the BOD POD for estimating percent fat in female college athletes. J. Strength Cond. Res..

[19] Bates D., Mächler M., Bolker B., Walker S. (2014). Fitting Linear Mixed-Effects Models Using lme4. arXiv.

[20] Lenth R. Emmeans: Estimated Marginal Means, Aka Least-Squares Means. R Package Version 1.4.5. https://CRAN.R-project.org/package=emmeans.

[21] R Core Team (2020). R: A Language and Environment for Statistical Computing.

[22] Wickham H. (2019). Welcome to the tidyverse. J. Open Source Softw..

[23] Wickham H., Bryan J. Readxl: Read Excel Files. R Package Version 1.3.1. https://CRAN.R-project.org/package=readxl.

[24] Kuznetsova A., Brockhoff P.B., Christensen R.H.B. (2017). lmerTest Package: Tests in LinearMixed Effects Models. J. Stat. Softw..

[25] Robinson D., Hayes A. Broom: Convert Statistical Analysis Objects into Tidy Tibbles. R Package Version 0.5.5. https://CRAN.R-project.org/package=broom.

[26] Binkley T.L., Daughters S.W., Weidauer L.A., Vukovich M.D. (2015). Changes in Body Composition in Division I Football Players Over a Competitive Season and Recovery in Off-Season. J. Strength Cond. Res..

[27] Delisle-Houde P., Reid R.E.R., Insogna J.A., Chiarlitti N.A., Andersen R.E. (2019). Seasonal Changes in Physiological Responses and Body Composition During a Competitive Season in Male and Female Elite Collegiate Ice Hockey Players. J. Strength Cond. Res..

[28] Suarez-Arrones L., Lara-Lopez P., Maldonado R., Torreno N., De Hoyo M., Nakamura F.Y., Di Salvo V., Mendez-Villanueva A. (2019). The effects of detraining and retraining periods on fat-mass and fat-free mass in elite male soccer players. PeerJ.

[29] Silva J.R., Brito J., Akenhead R., Nassis G.P. (2016). The Transition Period in Soccer: A Window of Opportunity. Sports Med..

[30] Booysen M.J., Gradidge P.J., Constantinou D. (2019). Anthropometric and Motor Characteristics of South African National Level Female Soccer Players. J. Hum. Kinet..

[31] Martinsen M., Sundgot-Borgen J. (2013). Higher prevalence of eating disorders among adolescent elite athletes than controls. Med. Sci. Sports Exerc..

[32] Andrews A., Wojcik J.R., Boyd J.M., Bowers C.J. (2016). Sports Nutrition Knowledge among Mid-Major Division I University Student-Athletes. J. Nutr. Metab..

[33] Plateau C.R., McDermott H.J., Arcelus J., Meyer C. (2014). Identifying and preventing disordered eating among athletes: Perceptions of track and field coaches. Psychol. Sport Exerc..

